# Modeling Transcuticular Uptake from Particle-Based Formulations of Lipophilic Products

**DOI:** 10.1021/acsagscitech.2c00029

**Published:** 2022-04-28

**Authors:** Joseph R. Elliott, Richard G. Compton

**Affiliations:** Department of Chemistry, Physical and Theoretical Chemistry Laboratory, University of Oxford, South Parks Road, Oxford OX1 3QZ, Great Britain

**Keywords:** foliar uptake, cuticle, diffusion, pesticide, formulation

## Abstract

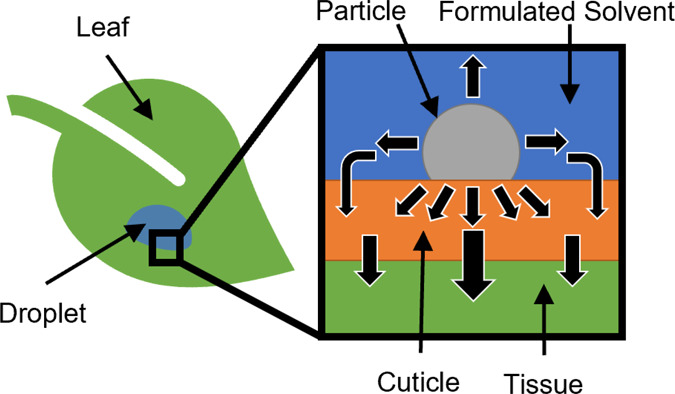

We report a mathematical model for the uptake of lipophilic agrochemicals from dispersed spherical particles within a formulation droplet across the leaf cuticle. Two potential uptake pathways are identified: direct uptake via physical contact between the cuticle and particle and indirect uptake via initial release of material into the formulation droplet followed by partition across the cuticle-formulation interface. Numerical simulation is performed to investigate the relevance of the particle-cuticle contact angle, the release kinetics of the particle, and the particle size relative to the cuticle thickness. Limiting cases for each pathway are identified and investigated. The input of typical physicochemical parameters suggests that the indirect pathway is generally dominant unless pesticide release is under strict kinetic control. Evidence is presented for a hitherto unrecognized “leaching effect” and the mutual exclusivity of the two pathways.

## 1 Introduction

The improved efficacy of application of agrochemicals to crops and weeds is vital to the future development of the agricultural industry.^1^ Pressure to reduce the agrochemical input in response to its ecological side-effects is increasing,^2−4^ while current methods have been demonstrated to be of very limited uptake efficacy.^5,6^ Commonly applied agrochemicals are pesticides, which include herbicides, fungicides, and insecticides.^7^

Pesticides are frequently sold as spray-applied formulations. Many categories are available. Of particular significance are dispersion-based formulations in which the pesticide, often poorly water-soluble, exists within the droplet as finely dispersed particles of approximately micrometer^8^ or sub-micrometer dimensions.^8,9^

The barrier to entry of pesticides of intermediate to high lipophilicity is the plant cuticle, a layer of cutinous polymer matrix and wax that covers the epidermal cells of most leaves and acts as a protective solubility and transport barrier.^10,11^ The cuticle has an inner “sorption compartment” and an outer “skin” layer, often referred to as the “cuticle proper”.^12−14^ The intracuticular wax within the cuticle proper often restricts diffusion to greatly tortuous paths^11,13−15^ and reduces diffusion. The cuticle proper is accepted as the limiting step to cuticular uptake.^13,14,16^ Although alternatives exist,^17,18^ this model is widely accepted and used in this study. Diffusion through the lipidic cuticle is the main lipophilic uptake route, rather than via stomata^19^ or hydrophilic pores.^20^

Enhancing the efficacy of foliar uptake of pesticides reduces use of an active ingredient (AI),^21^ generating both environmental and economic benefits. Accurate modeling and simulation of the processes involved with uptake are preponderant to the pursuit of improved efficacy.^22^ Various models have been proposed for diffusion of agrochemicals across the cuticular membrane, from simple empirical relationships^23−26^ to more complex computational models.^27−32^ The study of release of active ingredients from designed particles is also extensive, with the popular Higuchi,^33^ Ritger–Peppas,^34^ and other models,^35−40^ including those accounting for particle swelling,^41,42^ particle erosion,^43−46^ multi-layer particles,^47,48^ and burst release.^49,50^

There is no model known to the authors that accounts for simultaneous release of a pesticide from a particle and its diffusion across the cuticle. No other model accounts for the following: the hindrance of pesticide release from particles proximal to a barrier surface; discrete, localized sources rather than a homogeneous solution source; and competition between direct uptake into the cuticle and indirect uptake via diffusion through the solution medium. These interactions are of great importance to spray-applied particulate agrochemicals and particulate contaminants. Application of a model considering only one of these processes is only effective for limiting cases. Modeling release from non-spherical particles is often avoided in the field of controlled release from particles,^38^ leaving a dearth of knowledge. While Mercer^27^ and Tredenick et al.^28,30^ have considered truncated spheres on the cuticle boundary, their models are applied to saturated droplets rather than pesticide-carrying particles.

The following focuses on how the release of pesticide from particles, dispersed on the outer cuticular surface and surrounded by an aqueous medium, affects the overall diffusion of pesticide into and across the cuticle proper. We couple together the two modeling problems of diffusion across a barrier and release from a discrete particle in the context of foliar uptake into the cuticle proper. We address several key questions relevant to the overall mechanism of uptake and identify qualitative and quantitative trends for dispersed-particle formulations:

- How does release of the pesticide into the aqueous droplet, followed by partitioning into the cuticle proper, compete with release directly into the cuticle proper in terms of its contribution to the uptake?
- How does the release rate from discrete particles affect the uptake of pesticide under a zero-order kinetics release mechanism?
- How does the presence of a low permeability barrier affect zero-order release from and diffusion about a particle suspended in solution? How does the geometry of this system affect the transport behavior across such a barrier?
- Does the relative thickness of the cuticle proper affect the release from the particle and uptake under this simplified model?
- How does the particle-cuticle-aqueous contact angle affect the uptake for a truncated spherical particle?
- What limiting cases can be identified and how can we use these to understand the system?

These questions are answered in the Results and Discussion section along with relevant simulated results. A description of the computational model is provided first.

## 2 Theory

**Figure 1:**
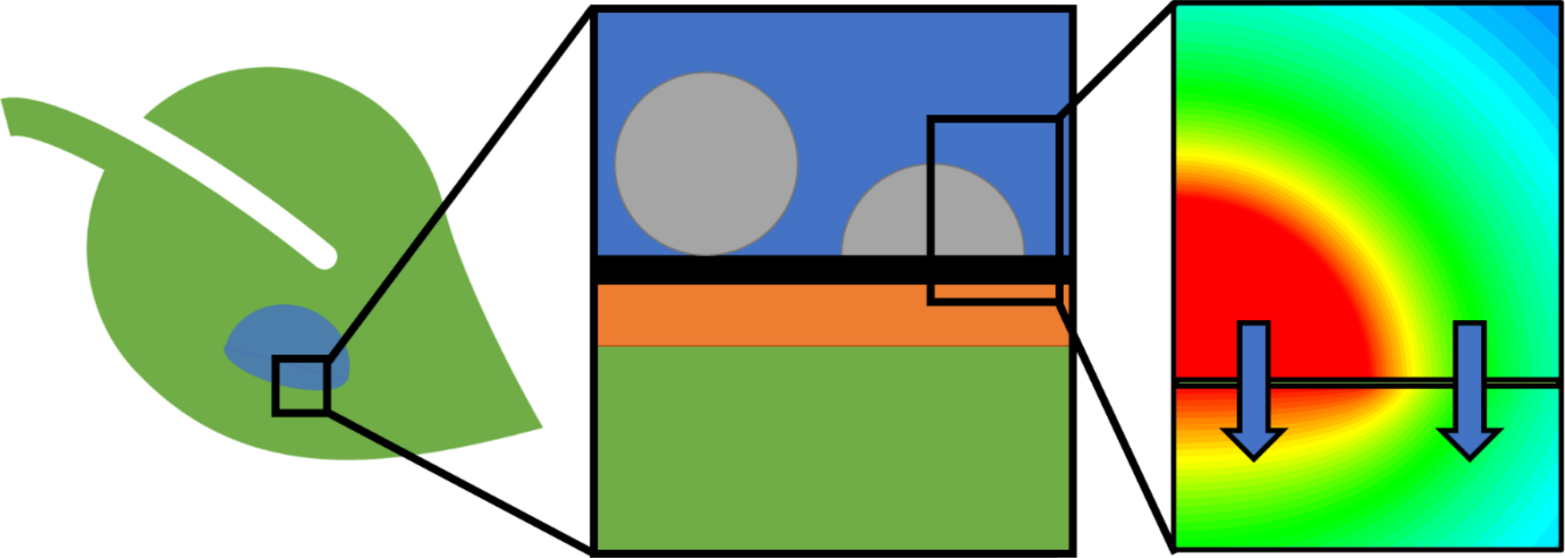
Schematic (not to scale) whereby particles in contact with the cuticle and within a droplet on a generic leaf surface release material either directly into the cuticle or into the aqueous medium prior to uptake through the leaf surface. The second panel provides a schematic of particles (gray) in the aqueous droplet (blue) on the cuticle surface (cuticle proper in black and sorption compartment in orange) with plant tissue represented by a green continuum. Fickian diffusion and partitioning across the cuticle-solution interface develop two possible pathways for uptake: directly through the particle-cuticle contact or indirectly via the cuticle-solution interface, represented by the blue arrows. The thick black line in the third panel represents the outer boundary of the cuticle proper. Illustrative steady-state concentration profiles developed by Fickian diffusion are provided as color maps in the third panel in the purely illustrative case of 1:1 partitioning.

We model pesticide uptake from a particle on the cuticle surface as occurring via two possible routes, which is illustrated in Figure 1: first, a direct pathway with release directly into the cuticle proper via particle-cuticle contact, followed by diffusion through the cuticle proper, and second, an indirect pathway with release into the surrounding solution, followed by diffusion through the aqueous medium, partitioning into the cuticle proper, and diffusion through the cuticle proper.

In this work, we assume that stomatal penetration is a negligible uptake pathway from the droplet.^31^ We further neglect penetration of adjuvant species into the cuticle for simplicity and treat transfer from the cuticle proper to the sorption compartment as much faster than entry to the cuticle proper; post-cuticular activity is beyond this study’s scope. We also neglect evaporation of the droplet to maintain simplicity; typical evaporation times are considered in the Results and Discussion. Convective currents and droplet edge effects are ignored. The pesticidal species is assumed to be neutral. These assumptions allow focus on the coupling of release from the particle with the diffusion across the cuticle barrier. Epicuticular waxes also affect uptake through their wetting properties^51,52^ and trapping of particulate material.^53−55^ However, as they have been demonstrated not to act as a transport barrier,^56−58^ these influences are outside the scope of this work.

Zero-order release and first-order re-absorption kinetics are applied at the particle interfaces according to eq 1
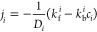
1where *i* represents the aqueous (aq) or cuticular (cut) media, *j_i_* is the diffusive flux (mol · m^–2^ · s^–1^) into medium *i*, *D_i_* is the diffusion coefficient within medium *i*, *k*_f_^*i*^ is the pesticide’s release rate constant into medium *i*, *k*_b_^*i*^ is the pesticide’s re-absorption rate constant from medium *i*, and *c_i_* is the surface concentration of pesticide within medium *i*. The simple release model given in eq 1 allows focus on the coupling between the diffusive transport and the interfacial kinetics. Transport is modeled as purely Fickian diffusion.^59^ A complete description of the model and solution methods is provided in Section 1 of the SI.

**Figure 2:**
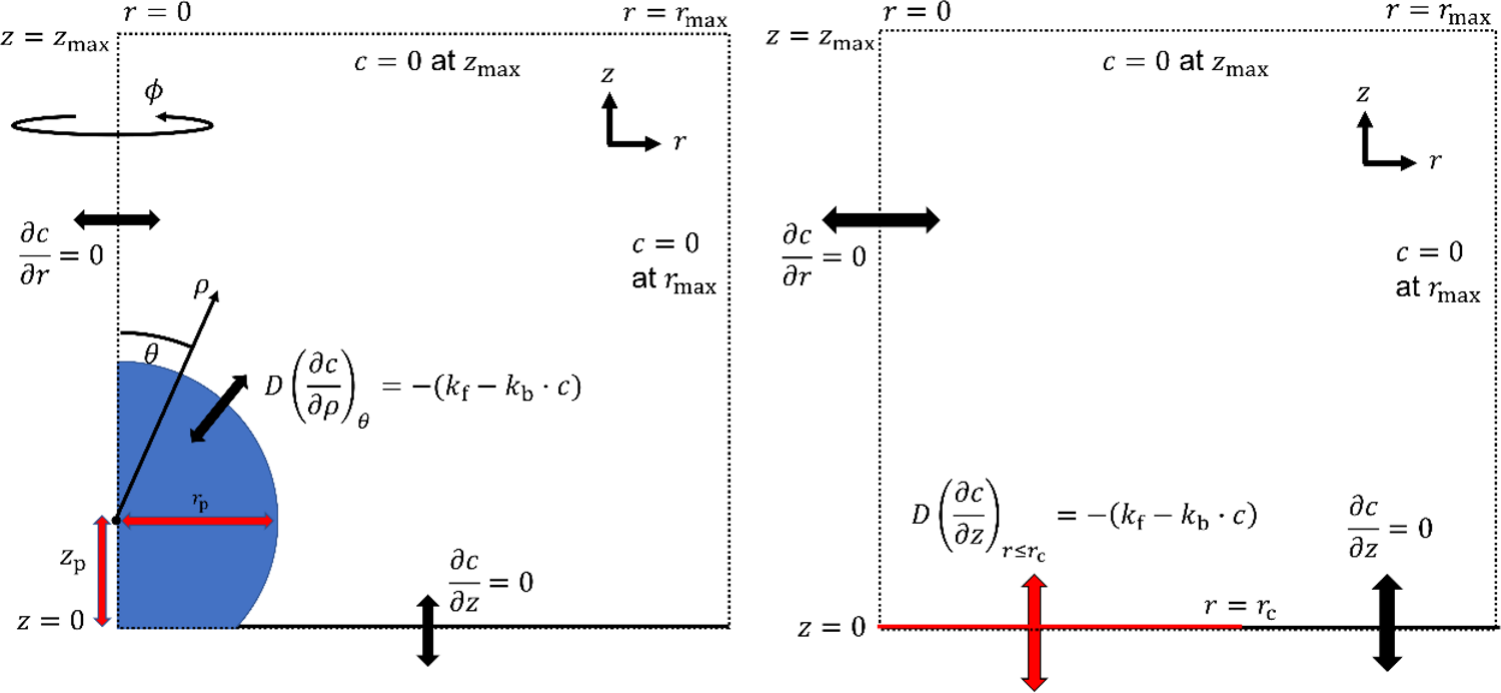
Illustration of model boundary conditions and coordinate systems.

Schematics illustrating the boundary conditions and coordinate systems used for the truncated sphere and disk models are shown in Figure 2.

Processes are added sequentially to the model, and simulation results are analyzed at each step. The order of processes introduced is as follows: unbounded release from a spherical particle; release from a truncated spherical particle constrained by an inert barrier; release from a circular particle-cuticle contact area into a slab of finite thickness; and surface-equilibrated partitioning of material between the aqueous and cuticle proper phases, with and without simultaneous release via particle-cuticle contact. The direct and indirect pathways are simulated individually and then in tandem. A benefit of the model’s format is the easy incorporation of additional processes and thus offers a solid physical foundation for further model development. Simulation results for these models are presented sequentially in the Results and Discussion.

Physical variables are converted into dimensionless forms to simplify and generalize the model.^60,61^ The conversions used are presented in Section 1.4 of the SI. The particles are modeled as (truncated) spheres, and cylindrical coordinates (*r*, *z*) are used to describe the system. [A]^*i*^ and [A]_eq_^*i*^ are the concentration and equilibrium concentration in medium *i*, *r*_p_ is the radius of the particle, and *D*_ref_ is the reference diffusion coefficient (=*D_i_* while media are simulated individually).

**Figure 3:**
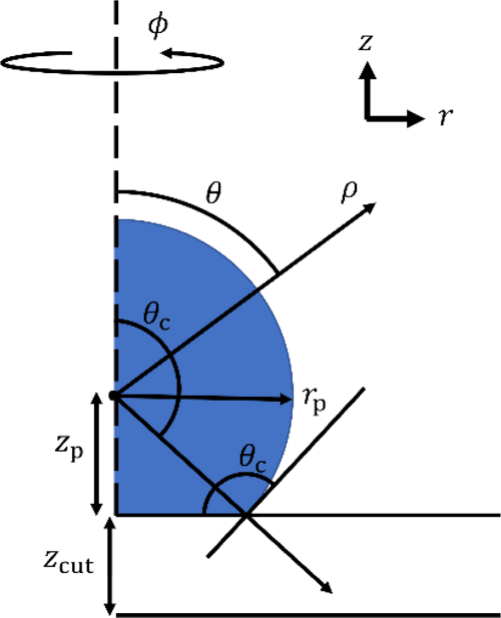
Schematic illustration of the spatial parameters describing a truncated sphere resting on a finite barrier under cylindrical coordinates (*r*, *z*). Parameters include the angle from symmetry axis θ, angle to three-phase contact point θ_c_ (equivalent to the contact angle), particle radius *r*_p_, shortest distance from particle center ρ, distance from particle center to barrier surface *z*_p_, and barrier thickness *z*_cut_.

*z*_p_ is the perpendicular distance from the cuticle proper to the particle’s center, and θ_c_ is the angle from the particle’s center to the contact point, which is equivalent to the particle-cuticle-solution contact angle. *z*_cut_ is the cuticle proper thickness. The model’s spatial parameters are illustrated in Figure 3.

Solution of Fickian diffusion within this model uses the ADI (alternating direction implicit) method^62^ after spatial and temporal discretization using the finite difference method.

Reliable simulation results must be converged and accurate.^63^ Steady-state simulations are performed iteratively until the total mass varied by <0.01% and spatially converged by total surface flux within 0.1%. Where analytical results are applicable, the total flux is accurate within 0.1%. The profiles of flux across the active surface are similarly accurate to literature. Time-dependent simulations use the same spatial grid. Flux-time profiles are accurate within 1% of literature where available and within 0.1% at long times. Time-dependent solutions have a total mass conservation error below 10^–5^%. Where analytical results are not available, comparison to known cases and assessment of the continuity of results are used for validation.

## 3 Results and Discussion

In this section, we present and discuss results from simulations of the above model.

### 3.1 Modeling Particle Release into an Infinite Medium

Results for a spherical particle in an infinite aqueous volume are within 0.1% agreement with the analytical expression derived by Crank,^64^ validating the simulation method. These results are available in Section 3 of the SI.

### 3.2 Modeling Particle Release at the Aqueous-Cuticle Interface

We next perform two-dimensional steady-state simulations of pesticide release into aqueous solution from truncated spheres supported on an inert surface. We consider how aqueous release is affected by the dimensionless aqueous release rate constant *K*_aq_ and the extent of truncation, parametrized by *Z*_p_ or θ_c_.

We first perform simulations using *K*_aq_ = 10^6^ ≫ 10^2^ such that the surface is equilibrated and the release is independent of *K*_aq_: the thermodynamic limit.

**Figure 4:**
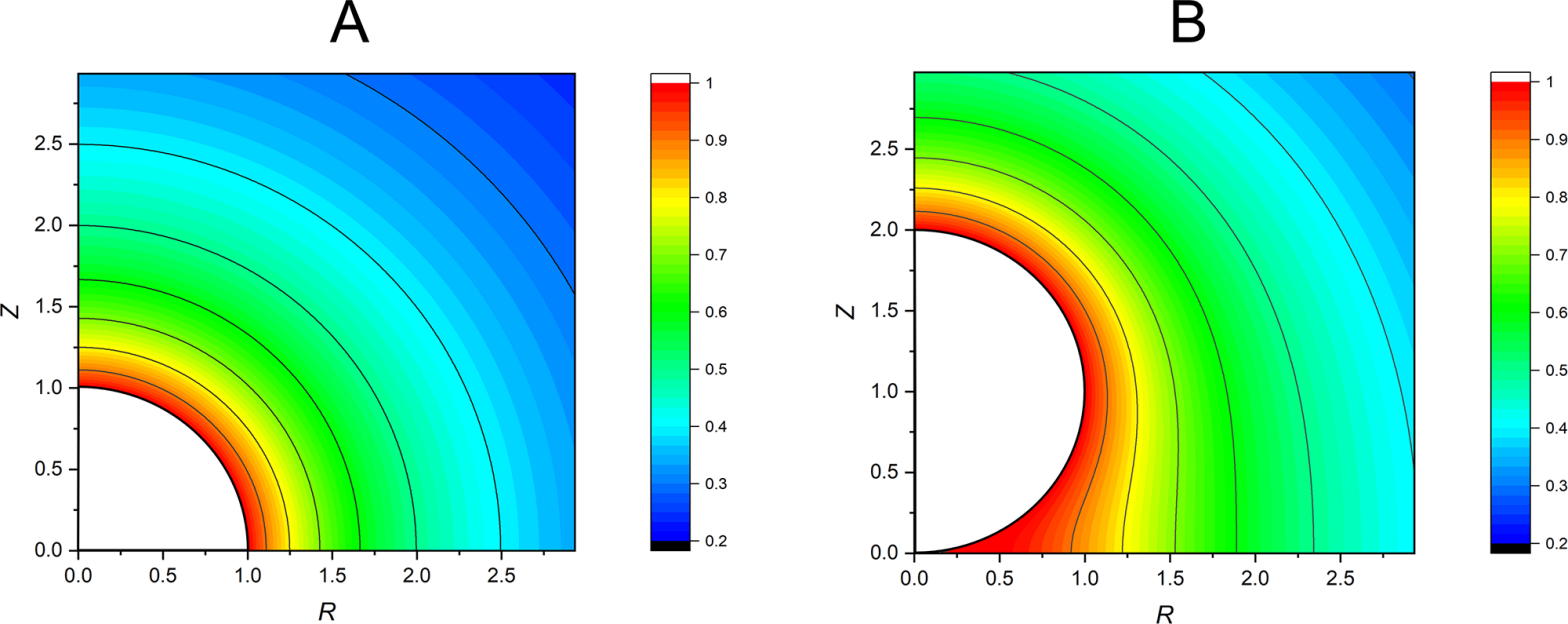
Dimensionless concentration profiles of aqueous, released material under the thermodynamic limit for (A) a hemisphere on a plane (*Z*_p_ = 0) and (B) a sphere on a plane (*Z*_p_ = 1) represented as color maps. The white area is the particle. The spatial dimensions are normalized to the particle radius. Isoconcentration contour lines are included.

**Figure 5:**
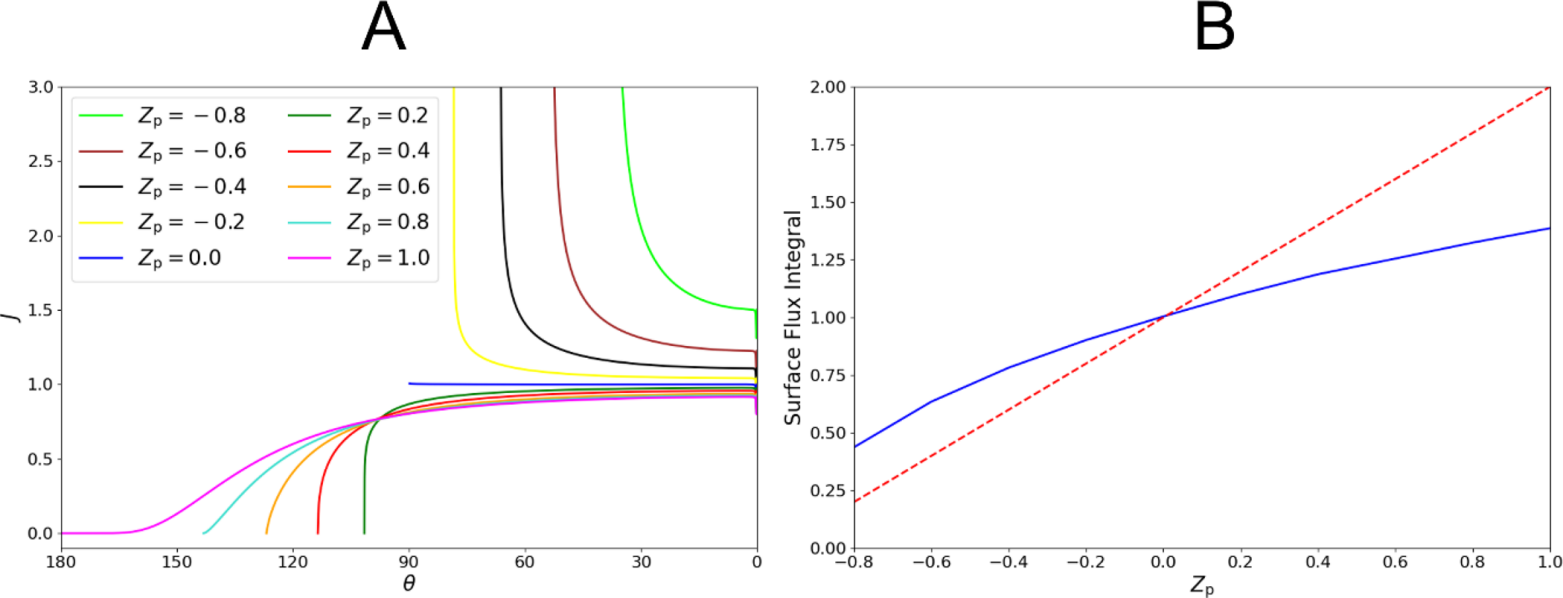
(A) Dimensionless steady-state flux profile for −0.8 ≤ *Z*_p_ ≤ 1. (B) Dimensionless steady-state surface flux integral ∫_0_^θ_c_^*J*(θ) sin θdθ = *J*_Tot_/2π for −0.8 ≤ *Z*_p_ ≤ 1, represented as a blue solid line, for a truncated sphere on a surface under thermodynamic release. The surface flux integral assuming a constant *J*(θ) = 1 is represented by a red dotted line in (B).

We consider the limiting cases of hemispherical (*Z*_p_ = 0) and spherical particles (*Z*_p_ = 1) on the surface with respect to their concentration profiles, which are presented in Figure 4. We also consider the dependence on *Z*_p_ of the dimensionless steady-state flux profiles *J*(θ) (Figure 5A) and the total dimensionless steady-state flux *J*_Tot_ as represented by the integral ∫_0_^θ_c_^*J*(θ) sin θdθ = *J*_Tot_/2π (Figure 5B).

The case of the hemisphere on a plane is isomorphic with half of an unbounded sphere. This is evident in Figure 4 as the concentration profile exhibits no θ-dependence and is spherically symmetric.

In contrast, the sphere on a plane does not give a θ-independent concentration profile. Figure 5A shows that the local flux is reduced at all points on the surface. The reduction becomes increasingly prominent closer to the contact point. This suggests that release is limited by geometrically hindered diffusion. A diffusionally stagnant zone develops between the spherical particle and the cuticular plane, causing a buildup of material, as seen in Figure 4. Some diffusional stagnation persists around the entire particle. Figure 5B demonstrates that the total flux deviates by a factor of ln2 ≈ 0.69 from that predicted for an isolated sphere. These results for the hemisphere and sphere are consistent with analytical and experimental results,^65−68^ providing validation to this model.

Examples 0 < *Z*_p_ < 1 exhibit a semi-stagnant zone of intermediate effect. As seen in Figure 5, the decay to *J*(θ) = 0 is sharp, becoming less sharp for larger *Z*_p_. The development of a stagnant zone for which *J*(θ) ≈ 0 occurs only for *Z*_p_ > 0.8. The diffusionally stagnation is observed as a negative curvature in the surface flux integral in Figure 5B. These results are consistent with analytical results for the total flux,^65^ validating the local flux profiles that are new to the literature.^68^

For *Z*_p_ < 0 or θ_c_ < 90^°^, diffusion from the sphere near the contact point is enhanced. This is an opposite phenomenon to that seen in the stagnant zone since the diffusionally accessible volume at the contact point is increased relative to the hemisphere case. This results in an enhanced local flux. The flux at the contact point tends toward infinity. This model presents novel total flux calculations for spherical caps of *Z*_p_ < – 0.4 as well as novel local flux profiles for *Z*_p_ < 0, due to the limitations of previous analytical approaches.^65^

The non-linearity of the dependence of the release on truncation is relevant to fast-release dispersion-based formulation design, such as pure pesticide particles (e.g., wettable powders, water dispersible granules, suspension concentrates, and oil dispersions),^69^ rapid burst release mechanisms,^49,50^ and triggered mechanisms.^70^ The correction to the total release rate needed for a given *Z*_p_ relative to a hemisphere is given in Figure 5B. Our model corrects the rate of aqueous release and gives the non-uniform release and concentration profiles. The stagnant zone close to the cuticle is highly pertinent to uptake across the cuticle-solution interface.

Reducing *K*_aq_ below 10^–2^ and entering the kinetic regime results in a uniform steady-state surface flux *J* = *K*_aq_ independent of the truncating surface. We can thus conclude that only particles with rapid release kinetics relative to diffusion exhibit deviations from unidimensional release models and benefit from tuning of the cuticle-particle contact angle. Simulation results illustrating the thermodynamic-kinetic regime transition under this geometry are given in Supplementary Figure 6.

### 3.3 Modeling Particle Release Directly into the Cuticle and the Effects of Cuticle Thickness, Release Kinetics, and Localization of the AI Source

In previous sections, we consider particle release into an aqueous phase. We next discuss direct transfer of pesticide into the cuticle proper. We approximate the area of particle-cuticle proper contact as a 2D disk through which uptake occurs exclusively. We simulate the release and diffusion from this disk contact across a barrier of finite thickness, *Z*_cut_ = *z*_cut_/*r*_p_, where *z*_cut_ and *r*_p_ are defined in Figure 3. Distinctions from the truncated sphere model are given in Section 2 of the SI. We treat the cuticle proper-sorption compartment interface as a perfect sink.

We first simulate various *K*_cut_ values, the dimensionless rate constant for release into the cuticle proper, with a barrier thickness in the limit of infinite thickness (*Z*_cut_ = 1000 ≫ 1), and identify thermodynamic and kinetic limits (Supplementary Figure 7). For the kinetic limit (*K*_cut_ ≤ 10^–2^), the steady-state flux is uniform across the disk: *J*(*R*) = *K*_cut_. The thermodynamic regime exhibits a steady-state surface flux accurate to the expression derived by Aoki.^71^ These results are validation for our model.

Diffusion at a disk into/from an infinite medium is well-described by the literature.^72^ However, the cuticle proper and particles are typically similarly sized, on the scale of micrometers to tens of nanometers.^9,73−75^ This leads to marked differences from infinite-volume treatments and assumptions of unidimensional diffusion.^29,76^

**Figure 6:**
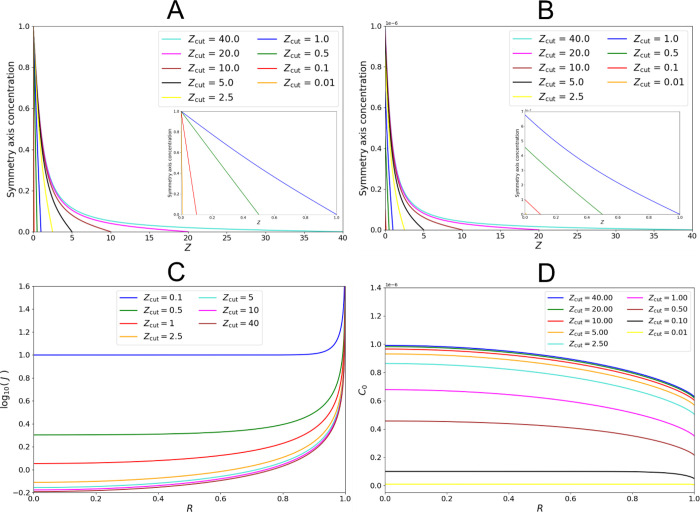
Results for the surface flux and cuticular concentration for varying *Z*_cut_ for a particle-cuticle disk interface. The steady-state concentration profile along the symmetry axis is presented for (A) thermodynamic release (*K*_cut_ = 10^6^) and (B) kinetic release (*K*_cut_ = 10^–6^). Both demonstrate transitions from linear diffusion. Inlaid diagrams provide clearer illustration for small *Z*_cut_. (C) presents the steady-state surface flux profile for varying *Z*_cut_ for *K*_cut_ = 10^6^. (D) presents the surface concentration profile for varying *Z*_cut_ for *K*_cut_ = 10^–6^.

We simulate varying *Z*_cut_ under the thermodynamic and kinetic regimes: the steady-state concentration profiles along the symmetry axis are given in Figure 6A and Figure 6B, respectively. The concentration profiles deviate from linearity as *Z*_cut_ increases, reflecting that diffusion in the *r*-direction increasingly contributes.

Figure 6C illustrates the effect of an increasing *Z*_cut_ on the steady-state surface flux under the thermodynamic regime. The release rate deviates from that for a linear concentration gradient: . An edge effect develops, which affects the local flux increasingly far from *R* = 1. The local surface flux increasingly resembles Aoki’s prediction,^72^ and the total flux decreases to *J*_Tot_/*r*_p_^2^ = 4.

Under the kinetic regime, the steady-state surface flux is unaffected by *Z*_cut_. Linearity of the concentration profile is always maintained. As *Z*_cut_ decreases, the concentration of pesticide within the cuticle proper decreases in order for the flux at the surface to equal both linear diffusion and the dimensionless release rate constant, i.e., . This can be seen in Figure 6B,D plots for *Z*_cut_ < 1.

For slow-release particles, this model predicts that application to a thinner cuticle proper or increasing the particle-cuticle contact area (using the contact angle or particle size) results in a lower pesticide concentration within the cuticle proper for the same uptake rate. This implies a reduced absorption of pesticide in the cuticle, a desirable formulation feature,^77−80^ so long as the direct uptake pathway is dominant.

For the case of diffusion across a finite, planar boundary, two pairs of limiting regimes exist: thermodynamic vs kinetic and infinite thickness vs constraining thickness. These regimes are summarized in Table 1. We use the total steady-state surface flux as the metric for determining the infinite-constrained transition under the thermodynamic limit and the thermodynamic-kinetic transition under the infinite limit, as it is stringent and most easily determined experimentally. The infinite-constrained transition for the kinetic limit is inferred from the steady-state concentration at the disk’s center. Plots illustrating these transitions are provided in Supplementary Figure 8. The flux at the perfect sink boundary is a potential metric for the successful penetration of material through the cuticle proper. The flux into the sorption compartment has a limiting case for small *Z*_cut_ of a step function from *J*(*R* ≤ 1) = 1/*Z*_cut_ to *J*(*R* > 1) = 0. As *Z*_cut_ increases, this flux becomes less localized. This is illustrated and discussed fully in Section 4 of the SI. Further work will assess potential effects of this localization of material on the transport beyond the cuticle proper. We expect that greater localization might produce steeper concentration gradients and a stronger driving force for uptake.

**Table 1:** Summary of the Four Limiting Cases alongside the Relevant Points of Transition

	thermodynamic limit	transition point	kinetic limit
infinite media limit	*C*_*Z* = 0_ = 1 for all *R*	*K*_cut_ = 4/*π* = 1.27	*C*_*Z* = 0_ = *f*(*R*)
	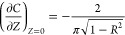		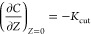
	non-linear diffusion		non-linear diffusion
	*J*_Tot_/*r*_p_^2^ = 4		*J*_Tot_/*r*_p_^2^ = π*K*_cut_
transition point	*Z*_cut_ = π/4		*Z*_cut_ = 1
constrained media	*C*_*Z* = 0_ = 1 for all *R*		*C*_*Z* = 0_ = *K*_cut_*Z*_cut_
	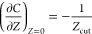		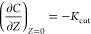
	linear diffusion		linear diffusion
	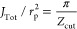		*J*_Tot_/*r*_p_^2^ = π*K*_cut_

It should be noted that smaller *Z*_cut_ results in a shorter time to attain steady-state diffusion within the cuticle proper (SI Section 5), which may inform controlled uptake models. These results demonstrate that greater contact area enhances direct uptake non-linearly: the area through which uptake occurs increases, the release kinetics accelerate (*K*_cut_ = *k*_f_^cut^ · *r*_p_/*D*_cut_ · [A]_eq_^cut^), and the *relative* barrier thickness *Z*_cut_ decreases. The flux is maximized in the thermodynamic, constrained regime. This non-linear dependence on area cannot be inferred from unidimensional or partition-limited models.

While an “effective” diffusion coefficient is used in our model, diffusion (and thereby transport) is treated here as homogeneous throughout the cuticle proper, which overly simplifies cuticles possessing highly tortuous structures^15,24^ or stratification of chemical components.^17^ Further work is required to assess the validity of these results in such cases. Large tortuosity values will complicate assessments of transport rates based on the cuticle proper thickness, as diffusion path lengths shall be greater.^15^ Use of volume-averaged *D*_cut_ and [A]_cut_^eq^ may be inaccurate for modeling despite their experimental utility.

### 3.4 Modeling the Indirect Uptake Pathway and Comparison to the Direct Pathway

We now explore the competition between release into solution and transport across the cuticle-particle interface. Particular attention is given to the pesticide solubility in aqueous formulation solution, [A]_eq_^aq^, the partition coefficient between aqueous formulation solution and the cuticle proper, *K*_cpw_, and the diffusion coefficient ratio, *D*_cut_/*D*_aq_.

We restrict our work to consider lipophilic pesticides and so approximate the solubility in the aqueous formulation solution as the aqueous solubility, [A]_eq_^aq^ ≈ [A]_eq_^H_2_O^, and approximate the aqueous-cuticle proper partition coefficient as log*K*_cpw_ ≈ – 1.108 + 1.01 log *K*_ow_,^81^ where *K*_ow_ is the octanol–water partition coefficient. We consider a *K*_ow_ range between 1 (e.g., mesotrione: 1.29) and 10^7^ (e.g., lambda-cyhalothrin).^82^ We thus consider a *K*_cpw_ range of 10^–1^ – 10^6^ and, similarly, an aqueous solubility range between 10^–7^ and 10^2^ mol/m^3^. Diffusion coefficients in water for small organic species are ∼10^–10^ m^2^ · s^–1^. Meanwhile, diffusion coefficients in the cuticle proper are comparable to diffusion coefficients in reconstituted wax^16^ ≈10^–18^ – 10^–17^ m^2^ · s^–1^. Diffusion through the cuticle proper is relatively very slow, reflecting the ratio *D*_cut_/*D*_aq_ ≈ 10^–8^ – 10^–7^.

Comparing the particle-solution and particle-cuticle interfaces, several possible regimes are identified for each interface: either thermodynamically or kinetically limited release with highly linear or non-linear diffusion dependent on *Z*_cut_. The interfacial fluxes also vary for different degrees of truncation.

We first consider the ratio of *Aj*_SS_(*i*) (where *A* is the interfacial area and *j*_SS_(*i*) is the dimensional steady-state flux at the interface between the particle and medium *i*) of a 1 μm radius particle that rests as a hemisphere on the cuticle proper (*Z*_p_ = 0), with *Z*_cut_ = 0.1.

If both interfaces are under the thermodynamic regime,
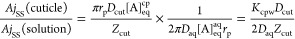
2

Considering typical values for lipophilic pesticides, we expect this ratio of interfacial fluxes to occupy a range between 5 × 10^–9^ and 5 × 10^–1^.

If the interface with the aqueous solution medium is kinetic and the interface with the cuticle proper is thermodynamic,
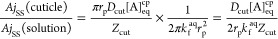
3where *k*_f_^aq^ is the forward release rate constant from the particle into the aqueous medium. If we approximate [A]_eq_^cp^ = [A]_eq_^H_2_O^ × *K*_cpw_, we shall expect this ratio of fluxes to vary between 5 × 10^–26^/*r*_p_*k*_f_^aq^ and 5 × 10^–9^/*r*_p_*k*_f_^aq^. For *r*_p_ = 1 μm, the expected range is 5 × 10^–20^/*k*_f_^aq^ to 5 × 10^–3^/*k*_f_^aq^.

If we consider *K*_cut_ = *k*_b_^cut^*r*_p_/*D*_cut_, in order for *K*_cut_ ≤ 1, for an *r*_p_ = 1 μm and *D*_cut_ = 10^–17^ m^2^ · s^–1^, *k*_b_^cut^ ≤ 10^–11^ m/s is required. Thus, we do not consider the cases in which the interface with the cuticle proper is under the kinetic regime since direct release is unlikely to be rate-limiting relative to diffusion through the cuticle proper.

We observe that the release of pesticide into the aqueous phase greatly outcompetes direct release into the cuticle proper unless the pesticide is simultaneously extremely lipophilic and poorly water-soluble, and the release into water is under kinetic control: in the most lipophilic and poorly water-soluble case within the ranges considered, *k*_f_^aq^ < 5 × 10^–3^mol · m^–2^ · s^–1^ is still required for direct release to be faster than indirect.

Though truncation does influence this ratio and must be considered for accurate measurement of the rates of release and uptake, only the most extreme scenarios might produce results in which release into the aqueous phase does not markedly outcompete direct release into the cuticle proper. As such, the influence of truncation on our broad assessment of the competition between these processes is neglected.

Both the direct and indirect pathways from the perspective of uptake into the leaf share a limiting step: diffusion-limited partitioning into the cuticle proper. As described above, diffusion is so much faster within the formulation that the steady-state aqueous concentration at the cuticle-solution interface is established instantaneously relative to the timescale of diffusion through the cuticle proper. By asserting mass balance at the interface, . This justifies the assumption that a concentration gradient that develops within the cuticle proper at the aqueous interface due to partitioning shall negligibly impact the concentration gradient within the formulation’s aqueous phase. With the dimensionless conversions, , using. We can thus reasonably approximate that the diffusion within the formulation’s aqueous phase is negligibly affected by the distribution of material in the cuticle proper.

Recognizing this, simulating the concentration profile along an inert cuticle-solution interface for a releasing truncated sphere and using this profile as a constant boundary condition at the cuticle-solution interface (assuming surface equilibration), we present a model for the diffusion of material across the cuticle proper, which occurs via the direct and indirect pathways, assuming zero initial/bulk concentration in the droplet and highly disperse particles.

**Figure 7:**
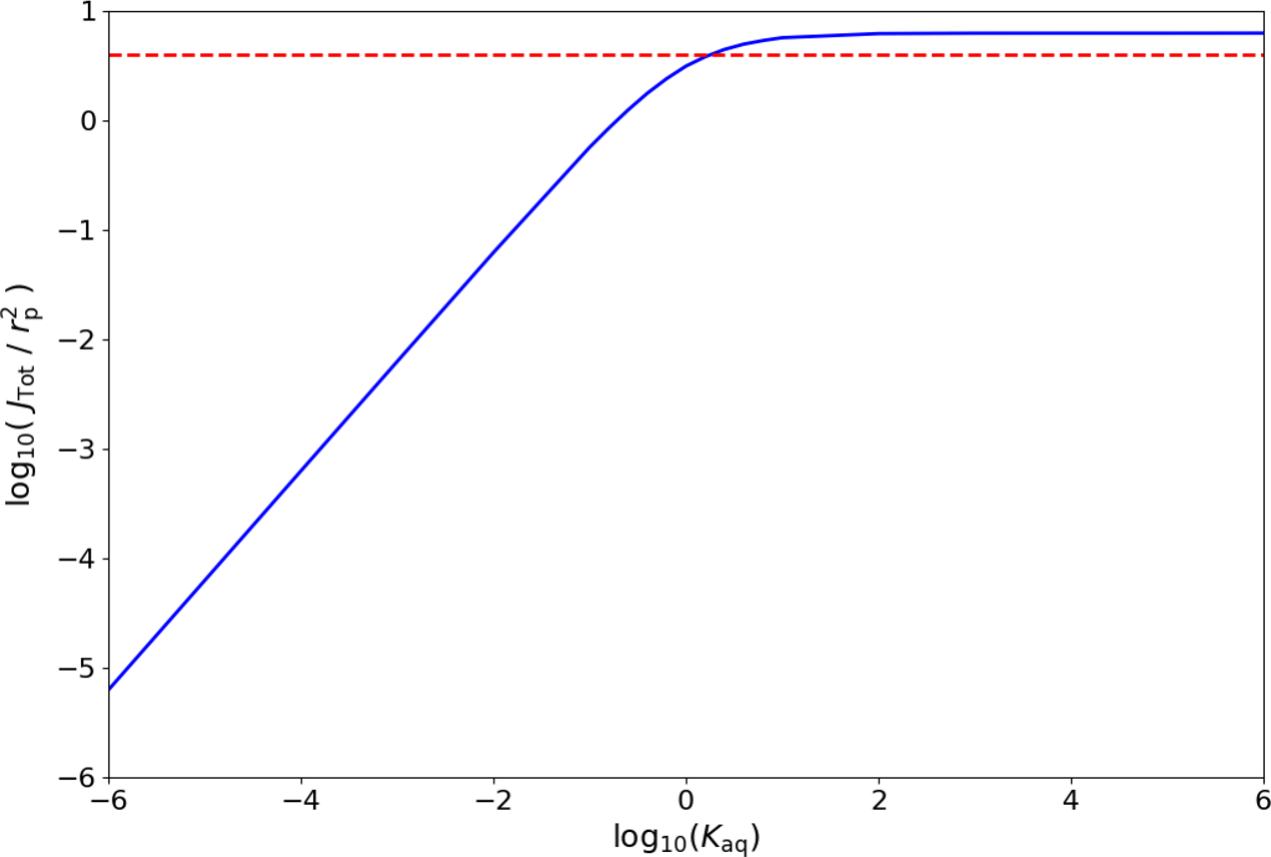
Logarithm of the dimensionless total steady-state flux *J*_Tot_/*r*_p_^2^ via the indirect pathway into an infinite cuticle (*Z*_cut_ ≫ 1) with zero direct flux from the disk contact area, assuming a surface-equilibrated cuticle-solution interface, a hemispherical particle (*Z*_p_ = 0), and instant repletion of material at the cuticle interface by diffusion through the aqueous solution for varying *K*_aq_. The red dotted line represents the dimensionless steady-state total flux predicted for the direct uptake rate acting alone under the thermodynamic, infinite-thickness regime.

Analysis of the direct pathway acting alone under the steady state is performed in Section 3.3. Consideration is now given to the case of the indirect pathway acting alone. The flux at the particle-cuticle interface is set to zero. The concentration along the solution-cuticle boundary is set as described above. Indirect uptake into the cuticle proper from a hemispherical particle is simulated for varying *K*_aq_ with diffusion through the cuticle proper under the infinite thickness regime and presented in Figure 7.

Comparison with the steady-state flux produced by direct uptake from a thermodynamic particle-cuticle interface of (*J*_Tot_/*r*_p_^2^ = 4) suggests that an aqueous dimensionless release rate constant of 1.8 or less is required for the indirect pathway to be slower than the direct pathway for the case of a hemisphere with *Z*_cut_ ≫ 1. SI Section 6 describes a factor, denoted *G*, which compares release rates from each interface to describe what regimes are available for uptake before complete depletion of the particle.

**Figure 8:**
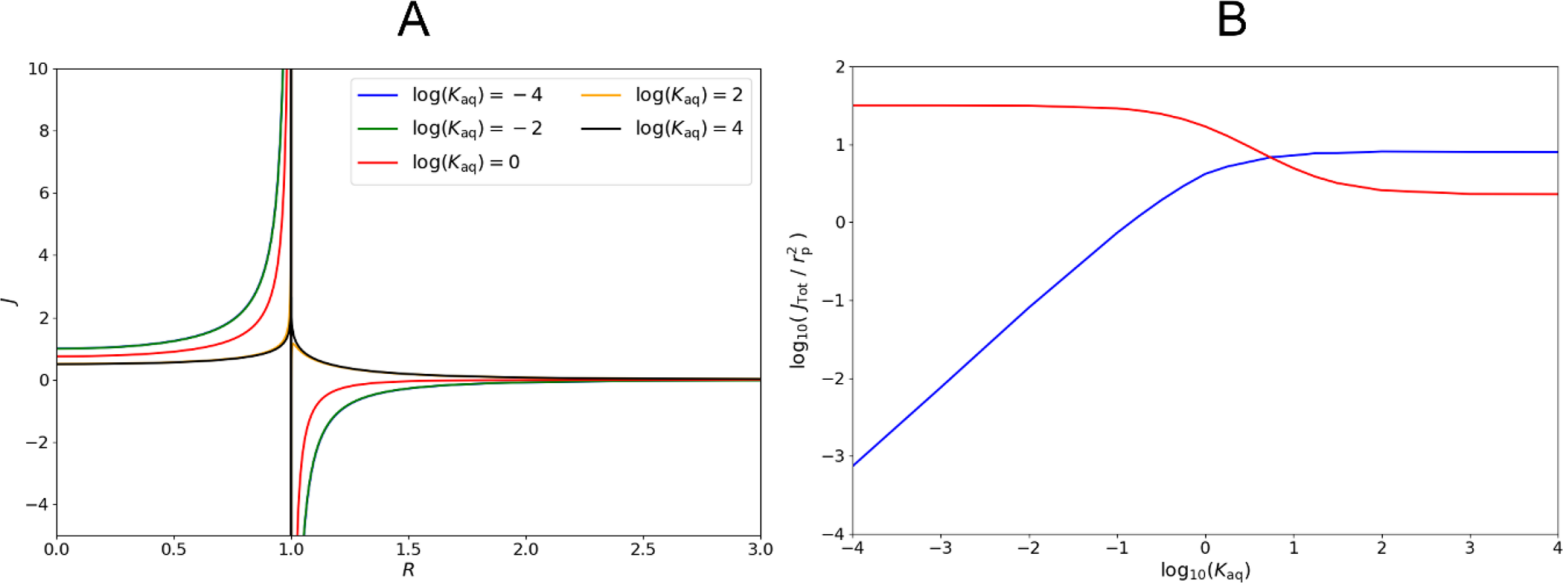
(A) Interfacial steady-state flux profile *J* for simultaneous direct and indirect uptake with varying dimensionless aqueous release rate constants, *K*_aq_. (B) Logarithm of the total dimensionless steady-state flux for simultaneous direct and indirect pathways into an infinite cuticle (*Z*_cut_ ≫ 1) assuming a thermodynamic cuticle-solution interface and instant repletion of material by diffusion through the solution for varying dimensionless particle-solution release rate constants *K*_aq_. The blue line represents the total flux into the cuticle. The red line represents the total flux into the cuticle directly from the particle. The difference between the two corresponds to the flux at the solution-cuticle interface (note that this is negative for *K*_aq_ < 10^0.8^).

If the two pathways are co-active, the total interfacial flux is not additive with respect to the indirect and direct uptake fluxes. In Figure 8, we present results of simulations performed under the assumptions described above with a cuticle-particle interface under thermodynamic control. The dimensionless aqueous release rate constant *K*_aq_ controls the rate of indirect uptake relative to direct uptake.

For high values of *K*_aq_, i.e., thermodynamic aqueous release, the two pathways compete. The resultant total flux approximates the indirect pathway acting alone, implying that direct uptake provides little benefit to fast-releasing particles and thus that models treating the whole droplets as homogeneous sources are accurate in these cases.

However, if the direct uptake outcompetes the indirect uptake, i.e., the particles are sufficiently slow-releasing, a negative interfacial flux is produced for *R* > 1, whereby material leaches from the cuticle proper back into aqueous solution, greatly hindering uptake across the cuticle proper so long as this alternative sink is available. This previously unrecognized leaching effect is of great potential significance as it suggests a dependence of the uptake of lipophilic material on the persistence of the aqueous medium, which has otherwise been discounted. This effect cannot be characterized by generally available models that rely on simple permeability relationships or partition-limited uptake. This effect requires experimental validation.

### 3.5 The Influence of Droplet Drying on the Uptake Timeframe

Evaporation of solvent from the formulation droplet influences pesticide uptake,^83^ and droplet drying is an obstacle to pesticidal uptake.^84^ Evaporation shrinks the droplet size and concentrates the dissolved active ingredient. This eventually results in crystallization or deposition of the active ingredient onto the outer cuticular surface. Modeling has been reported.^28,30,85^ Total evaporation of the droplet results in a (possibly hydrated) solid residue. Lipophilic species can continue to undergo uptake; however, at this stage, the rate of uptake is slowed and the mechanism by which the material continues to enter the cuticle proper is obscured. Our results in Section 3.4 demonstrate a leaching effect for slowly releasing particles, which also complicates the dependence of lipophilic uptake on the solvent evaporation. Consideration needs to be given to the evaporation time relative to the rates of uptake predicted within our model to assess the relevance of the indirect pathway and the leaching effect.

Calculations were performed whereby the times taken for a particle to fully deplete under a given steady-state surface flux were found for release into the aqueous phase and release directly into the cuticle proper
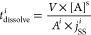
4where *V* is the particle volume, [A]^s^ is the density of the pesticide within the particle (mol · m^–3^), *A^i^* is the area of the particle exposed to medium *i*, *j*_SS_^*i*^ is the dimensional steady-state flux into medium *i*, and *t*_dissolve_^*i*^ is the time taken to fully deplete the particle by release into medium *i*, assuming constant steady-state flux.

For solid lipophilic pesticide particles, with densities varying between 0.7 and 10 mol · dm^–3^, it is found that 10^–2^ s ≤ *t*_dissolve_^aq^ ≤ 10^8^ s and 1 s ≤ *t*_dissolve_^cut^ ≤ 10^7^ s under the thermodynamic regime. If the particle-solution interface is under the kinetic regime, the corresponding result is . These calculations assume rapid attainment of the steady-state interfacial flux and neglects particle shrinking and moving boundary conditions.

The evaporation time of typical aqueous formulation droplets of 0.01 – 0.1 μL is typically on the order of 10^2^ – 10^3^ s.^86,87^ Our results suggest that direct uptake from solid particles outlasts the evaporation time significantly even for lipophilic species; thus, we predict the general persistence of lipophilic pesticide deposits on the outer cuticular surface long after evaporation if direct uptake dominates. The indirect pathway thus provides a means of accelerating uptake within this evaporation time, either by acting as an additional route into the cuticle or by reducing the leaching effect. It is reasonable to assume that a form of direct uptake is the dominant pathway after droplet evaporation.

Further work is required to assess how the leaching effect inferred from this model affects the overall uptake in these cases and whether a transition is observed if this leaching is removed by the solvent’s evaporation. Persistence of the aqueous droplet may prevent uptake for certain slow-release formulations. The model presented in this work in its current form is thus best applied to dispersed-particle formulations before evaporation of the solvent completes.

## 4 Comparison with Other Models

Our model builds upon unidimensional models that implicitly assume linear diffusion^16,29^ by demonstrating that this assumption is inaccurate for many modern application methods. Sufficiently small particle sizes are now in commercial use that the contact area radius through which uptake occurs can no longer be generally assumed to be much greater than the cuticle or cuticle proper thickness. This is crucial for slow-release, micro- and sub-microparticulate formulations for which uptake does not occur through the entire droplet area, illustrating this model’s impact. Numerous other models demonstrate the importance of multi-dimensionality for the simulation of other aspects relevant to transcuticular uptake including the influence of droplet shape and evaporation,^27,30,85^ air-cuticle uptake for semi-volatile ingredients,^31^ and characterization of diffusion about cuticular features.^32,88^

Additionally, several uptake models assume that the transport processes are partition-limited,^76^ including several mass-balanced multi-compartment models.^79,89^ Our work demonstrates the hitherto unrecognized importance of treating the particulate suspension and suspending solution as separate components in order to account for the competing direct and indirect pathways into the cuticle proper and the leaching effect that we observe. Additionally, this is significant in the consideration of differential uptake rates before and after droplet evaporation. Relevant corrections to partition-limited models have been illustrated in this work in order to consider the kinetics of the particulate release, the particle-cuticle contact angle, and the cuticle proper thickness relative to the particle-cuticle contact area.

While our model does not offer a complete estimation of uptake, it is nonetheless useful for improving estimates of uptake into and across the cuticle proper layer for larger whole-plant models of organic uptake and distribution.^79,89−91^ We expect that the use of our simulation results as relevant corrections to existing models will be impactful and useful in improving the accuracy of applying such models to particulate formulations.

## Supporting Information Available

Additional description of the model theory, numerical methodology, testing and validation procedures, results for a spherical particle releasing material into an infinite volume, discussion of the use of the far boundary flux as a metric for uptake, discussion of a parameter to predict the release behavior regime, and supplementary figures (general schematic for the different media and processes active during uptake of pesticide from a formulation droplet, illustration of spatial grid discretization schemes used, further results for an isolated spherical particle releasing pesticide into an unbounded medium, dimensionless steady-state flux profiles at the far boundary, logarithm of the total flux *J*_Tot_ from a disk into a finite planar barrier under the thermodynamic regime (*K*_cut_ = 10^6^) with a measure of time taken to deviate from the infinite volume result, the logarithm of the dimensionless steady-state flux profile for different values of *K*_aq_ for a full sphere on a surface, steady-state surface flux profile *J* across the particle-cuticle contact disk with *Z*_cut_ = 1000 and varying *K*_cut_, and illustration of direct uptake from a particle-cuticle disk contact area) and table (table of conversions from dimensional parameters to dimensionless parameters) (PDF)
