# Supplementary material for: Modeling Transcuticular Uptake from Particle-Based Formulations of Lipophilic Products
Source: ACS Agric Sci Technol. 2022 Apr 28;2(3):603–14. doi: 10.1021/acsagscitech.2c00029 (PMC9214695; doi:10.1021/acsagscitech.2c00029)
Supplement: Supplementary file 1 — as2c00029_si_001.pdf [file as2c00029_si_001.pdf]

Modelling Transcuticular Uptake from Particle-Based Formulations of Lipophilic Products

Supplementary Material

*Joseph R Elliott[1], Richard G Compton[1]**

[1]Department of Chemistry, Physical and Theoretical Chemistry Laboratory, University of Oxford, South Parks Road, Oxford, OX1 3QZ, Great Britain

*** Correspondence:** Richard G Compton; richard.compton@chem.ox.ac.uk

1 Theory

In this work, we simulate release from a particle on the cuticle surface, assuming a truncated spherical particle and representing the cuticle surface as an infinite slab of finite thickness. Truncation of the sphere by the plane is used to simulate variable physical contact between the particle and cuticle surface. We simulate the release from the particle into the cuticle through the circular contact area and approximate a sharp boundary between the 'cuticle proper' and the 'sorption compartment', which is further approximated to act as a perfect sink due to its greater solubility and diffusion coefficient.

Supplementary Figure 1 illustrates the formulation-leaf system and the simplifying approximations taken. The media involved are as follows: (1) the solid particle (2) the aqueous formulation droplet, (3) the 'cuticle proper' layer, (4) the 'sorption' layer of the cuticle, (5) the apoplastic leaf tissue, and (6) an open stoma in the leaf surface. The plausibly relevant processes are: (A) release of pesticide from the particle into solution, (B) release of pesticide from the particle into the 'cuticle proper', (C) partitioning of pesticide from solution into the 'cuticle proper', (D) partitioning of pesticide from the 'cuticle proper' into the 'sorption' layer of the cuticle, (E) partitioning of pesticide from the 'sorption' layer of the cuticle into the apoplastic leaf tissue, (F) vaporization of volatile pesticide from the formulation droplet, (G) uptake of pesticide into open stomata as a vapor, (H) evaporation of water from the formulation droplet, and (I) partitioning of accelerator adjuvant from solution into the 'cuticle proper'.

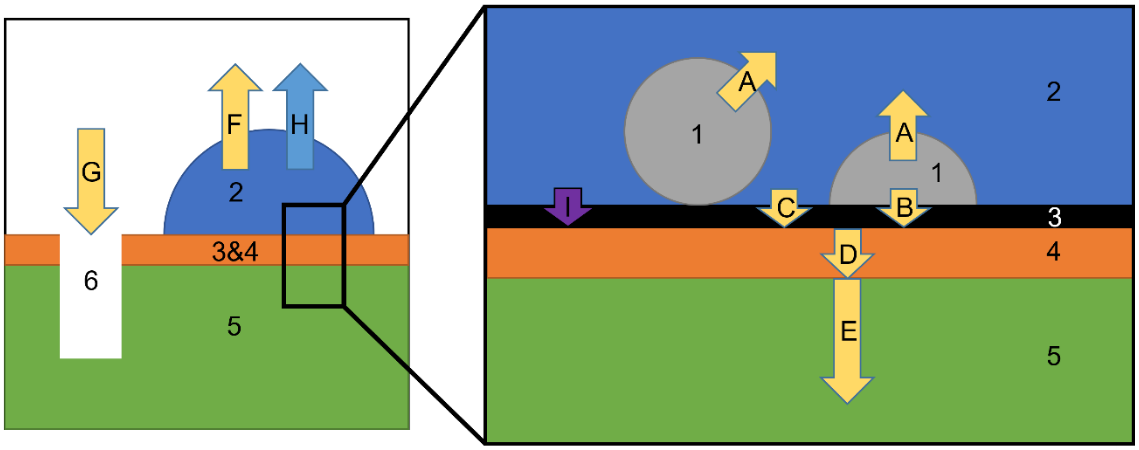

Supplementary Figure 1: General schematic for the different media and processes active during uptake of pesticide from a formulation droplet. The different media are labelled by number while the different processes are represented as arrows and labelled by letter. Explanation of these labels is given in the main text. Not to scale.

The processes E-I are neglected within the current model for simplicity as justified in the main text. While the effect of solvent evaporation H is not directly considered in the current model, a comparison of uptake times to evaporation times is given in the main text.

1.1 Fick's 2nd Law in cylindrical coordinates

The modelling of Fickian diffusion through a medium requires the solution of the partial differential equation(s) given by Fick's 2nd Law[1]:

$$\frac{\partial c}{\partial t} = \nabla \cdot (D \nabla c) = D \nabla^2 c \qquad \text{[Equation 1]}$$

where t is time, c is concentration and D is the diffusion coefficient assumed to be constant for a given medium.

The truncated sphere and disk models both possess an axis of symmetry which passes through the plane and sphere, so Fick's 2nd Law in cylindrical coordinates (r, z, ϕ) is

$$\frac{\partial c}{\partial t} = D \left[\frac{\partial^2 c}{\partial z^2} + \frac{\partial^2 c}{\partial r^2} + \frac{1}{r} \frac{\partial c}{\partial r} \right] \qquad \text{[Equation 2]}$$

since $\frac{\partial c}{\partial \phi} = 0$ at all ϕ, by symmetry.

To produce simulations of the steady state, we simplify Equation 2 further by using $\frac{\partial c}{\partial t} = 0$, since the concentration at any point is independent of time.

1.2 Fick's 2nd Law in spherical coordinates

For the simulation of a sphere in solution, with no proximal planar surface, spherical symmetry is observed. In this geometry,

$$\frac{\partial c}{\partial t} = D\left[\frac{\partial^2 c}{\partial \rho^2} + \frac{2}{\rho}\frac{\partial c}{\partial \rho}\right] \quad \text{[Equation 3]}$$

where ρ is the radial distance from the centre of the sphere.

1.3 Boundary conditions

Different types of boundary conditions are employed in this model.

A zero-flux boundary condition, described by $\frac{\partial c}{\partial x} = 0$, where x is a coordinate perpendicular to the boundary surface, is used at the axis of symmetry[2] and at the supporting surface in initial models.

A constant concentration boundary condition of $c(r^*, z^*) = c^*$ is applied at the boundary to the perfect 'sink' zone ($c^* = 0$) and at the boundary at which the solution becomes bulk ($c^* = 0$). The distance at which this condition can be accurately imposed is dependent on the expected root mean square distance travelled by a diffusing molecule ($\langle x \rangle_{rms} = \sqrt{6Dt}$ for a diffusing molecule in 3-dimensions as predicted by Brownian motion[3, 4]) and whether convective or other transport processes are active. Assuming negligible non-diffusional mass transport and a constant bulk concentration, we impose a constant concentration condition at a distance of $6\sqrt{Dt_{max}}$, far further than the expected distance diffused, where t_{max} is the maximum duration of the simulation.

An active boundary is one where $\frac{\partial c}{\partial x} = f$. This condition is used to describe the release process simply as $f = -\frac{1}{D}(k_\mathrm{f} - k_\mathrm{b}c)$, as described in the main text.

In the truncated sphere model, $x = \rho$ rather than r or z. By changing the variables used, the boundary condition is described as:

$$\left(\frac{\partial c}{\partial \rho}\right)_\theta = \left(\frac{\partial c}{\partial r}\right)_z \left(\frac{\partial r}{\partial \rho}\right)_\theta + \left(\frac{\partial c}{\partial z}\right)_r \left(\frac{\partial z}{\partial \rho}\right)_\theta = \left(\frac{\partial c}{\partial r}\right)_z \sin\theta + \left(\frac{\partial c}{\partial z}\right)_r \cos\theta \quad \text{[Equation 4]}$$
$$= \frac{1}{\rho}\left(r\left(\frac{\partial c}{\partial r}\right)_z + z\left(\frac{\partial c}{\partial z}\right)_r\right)$$

since $r = \rho\sin\theta$, and $z = \rho\cos\theta$.

A boundary condition is also required in the temporal dimension. The initial concentration at each point is set to the bulk solution, assuming no initial material in solution.

1.4 Dimensionless conversion

Simplification of Fick's 2nd Law and the boundary conditions is achieved by converting the parameters into dimensionless forms. This has the benefit of generalizing the solutions of these dimensionless problems[2]. The dimensionless conversions are provided in Supplementary Table 1. $[A]_\mathrm{eq}^i$ is the saturation concentration in medium i, D_ref is the reference diffusion coefficient, and r_p is the radius of the sphere for the truncated sphere model or the radius of the disk for the disk model.

Supplementary Table 1. Table of conversions from dimensional parameters to dimensionless parameters

Dimensional Parameter	Conversion to dimensionless form
Concentration, $[A]^i$	$C_i = [\mathrm{A}]^i/[\mathrm{A}]^i_{\mathrm{eq}} = k^i_{\mathrm{b}} \cdot [\mathrm{A}]^i/k^i_{\mathrm{f}}$
Diffusion coefficient, D_i	$d_i = D_i/D_{\mathrm{ref}}\,(= 1)$
Radial distance, r	$R = r/r_{\mathrm{p}}$
Distance from cuticle outer boundary, z	$Z = z/r_{\mathrm{p}}$
Thickness of the 'cuticle proper', z_{cut}	$Z_{\mathrm{cut}} = z_{\mathrm{cut}}/r_{\mathrm{p}}$
Distance from cuticle outer boundary to the particle's center, z_{p}	$Z_{\mathrm{p}} = z_{\mathrm{p}}/r_{\mathrm{p}}$
Time, t	$T = D_{\mathrm{ref}} \cdot t/r_{\mathrm{p}}^2$
Release rate constant into medium i, k_f^i<br>Re-adsorption rate constant from medium i, k_b^i	$K_i = k^i_{\mathrm{f}} \cdot r_{\mathrm{p}}/D_{\mathrm{ref}} \cdot [\mathrm{A}]^i_{\mathrm{eq}} = k^i_{\mathrm{b}} \cdot r_{\mathrm{p}}/D_{\mathrm{ref}}$
Surface Flux at a point into medium i, j_i	$J_i = j_i \cdot r_{\mathrm{p}}/D_{\mathrm{ref}} \cdot [\mathrm{A}]^i_{\mathrm{eq}} = j_i \cdot k^i_{\mathrm{b}} \cdot r_{\mathrm{p}}/(D_{\mathrm{ref}} \cdot k^i_{\mathrm{f}})$

Applying the dimensionless conversions to Fick's 2nd Law produces:

$$\frac{\partial C}{\partial T} = \frac{\partial^2 C}{\partial Z^2} + \frac{\partial^2 C}{\partial R^2} + \frac{1}{R}\frac{\partial C}{\partial R} \qquad \text{[Equation 5]}$$

The boundary at the particle interface condition is converted to:

$$\frac{\partial C}{\partial Z} = -K_{\mathrm{dissolve}}(1 - C) \text{ for the disk model} \qquad \text{[Equation 6]}$$

$$R\left(\frac{\partial C}{\partial R}\right)_Z + Z\left(\frac{\partial C}{\partial Z}\right)_R = -K_{\mathrm{dissolve}}(1 - C) \text{ for the truncated sphere model} \qquad \text{[Equation 7]}$$

1.5 Numerical Methods

The central three-point approximation is used for second order spatial derivative functions and the second-order central difference approximation is used for the first order spatial derivative functions. At the inner and outer boundaries, a forward or backward difference approximation is used for the spatial derivatives. Time discretization is achieved by backward difference approximation.

Expanding grids[5, 6] are used to ensure a high density of points within a certain region while minimizing the total number of points used for the simulations. Specifically, exponentially expanding grids are used according to Equation 8:

$$X_{i+1} - X_i = dX \times \omega_X^i = (X_i - X_{i-1})\omega_X \qquad \text{[Equation 8]}$$

where $0 \le i < n-1$ describes the point label, dX describes the initial step distance, and ω_X is the expansion factor in the X-direction. Illustrations of the grid schemes used are given in Supplementary Figure 2 for the different model geometries.

The truncated sphere model scheme should accurately describe the curved spherical surface and provide a high density of points around the spherical surface. We use the following scheme, introduced by Ward et al[7], to describe this problem (Supplementary Figure 2A). A series of points on the surface of the sphere are distributed uniformly along the θ-coordinate (each separated by an angle $d\theta$). The R- and Z-grids are then set to contain these points. This describes the density of points around the sphere using one parameter ($d\theta$). The grid is then expanded exponentially beyond $R = 1$ and $Z = 1 + Z_\mathrm{p}$.

When the sphere is truncated, a greater density of points is required for accurate simulation below $Z = Z_\mathrm{p}$. In this case, a second, smaller $d\theta_2$ is used between $\pi - \theta_\mathrm{s} \le \theta \le \theta_\mathrm{s}$ if $\left(\frac{\pi}{2}\right) < \theta_\mathrm{s} < \pi$, where θ_s is the θ value at which the sphere and inert surface make contact. This is seen in Supplementary Figure 2B, where the θ illustrated lies on the boundary between the two different θ-spacing regimes.

For the planar disk model, the Z-coordinate is discretized using an exponentially expanding grid. The R-coordinate requires a high density of points at the axis of symmetry and at the disk edge. The grid scheme expands and contracts such that $R = 0$ and $R = 1$ act as points around which the point density is maximized. For a low value of Z_max, the Z-grid contracts for $Z \ge \frac{Z_\mathrm{max}}{2}$ to give a well-defined outer boundary. The expansions and contraction use the same ω_R factor.

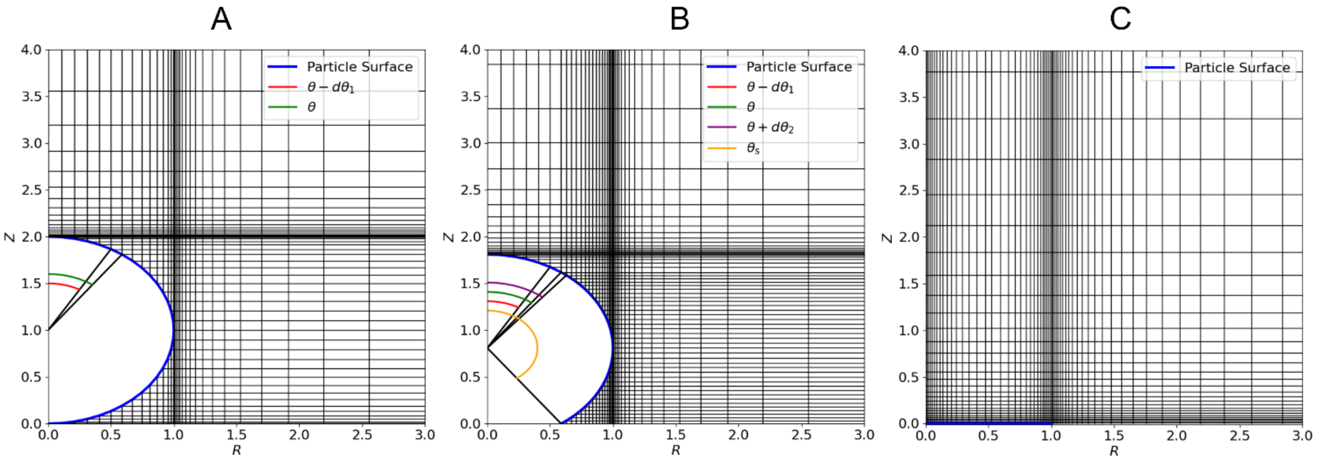

Supplementary Figure 2: Illustration of spatial grid discretization schemes used in this work for: (A) the truncated sphere model when $Z_\mathrm{p} = 1$ (illustrated with $d\theta = \frac{\pi}{30}$ and $\omega_R = \omega_Z = 1.3$) noting that the same scheme is used for $Z_p \le 0$, (B) the truncated sphere model when $0 < Z_\mathrm{p} < 1$ such that two θ-spacing regimes are used (illustrated with $Z_p = 0.8$, $d\theta_1 = \frac{\pi}{30}$, $d\theta_2 = \frac{\pi}{60}$, and $\omega_R = \omega_Z = 1.3$), and (C) the disk model (illustrated with $dR = dZ = 0.01$ and $\omega_X = \omega_R = 1.15$). The use of contraction in the z-direction for $Z > \frac{Z_\mathrm{max}}{2}$ in small Z_max regimes is not illustrated in (C). Discretization parameters chosen for this illustration are purely illustrative.

For the one-dimensional simulation of an isolated sphere in solution, one exponentially expanding spatial grid is required only in the ρ-coordinate.

An exponentially expanding grid is applied in the T-dimension, as the greatest change in concentration occurs when the active surface is first exposed to the medium and decays as the concentration profile approaches the steady state solution.

To generate a time-dependent solution, the PDE is solved for each time step sequentially. The ADI method [8-10] divides each time-step into two halves. During the first half of the time-step, the derivative functions in one spatial direction are discretized implicitly, while the derivative functions in the perpendicular direction are discretized explicitly. During the second half of the time step, the direction in which the derivatives are solved implicitly is swapped with the explicit direction. Thus, each coordinate receives equal parts implicit and explicit discretization. This methodology is efficient while providing guaranteed stability.

While the ADI method is useful for generating a time-dependent solution, it has also been used as an iterative process to converge upon (quasi-)steady-state solutions when $\frac{\partial c}{\partial t} = 0$. In this case, each application of the ADI process results in an additional iteration towards the steady-state solution rather than resolving a discrete step in time. ADI iterations are repeated until the concentration at each point in space changes by less than $10^{-2}\%$.

1.6 Software used

The simulations were performed using bespoke home-written *C++* programs with *OpenMP* used for parallel computing. The plotting of results was carried out in *Python* with the packages *NumPy*, *SciPy* and *matplotlib*. The plotting of 2D contour plots of concentration was carried out in *Origin 2020*.

1.7 Testing

Convergence testing was performed to find appropriate values for the parameters describing the discrete grid used in our simulations such that the results were independent of these values while minimizing simulation time. For the disk model, these parameters are dZ, ω_Z, dR, ω_R, dT, and ω_T. For the truncated sphere model, these parameters are $d\theta$, $(d\theta_2)$, ω_Z, ω_R, dT, and ω_T. For the truncated sphere, a convergence test must be performed for each Z_p value.

Results from these convergence tests were compared to analytical results where possible. The steady-state, diffusion-limited flux from a planar disk surrounded by inert surface in dimensionless coordinates is described analytically by [11] as

$$J = \frac{2}{\pi\sqrt{1 - R^2}} \qquad \text{[Equation 9]}$$

Meanwhile Shoup and Szabo[12] provide an empirical, approximate expression for the total diffusion-limited flux in the regime where significant convergent diffusion acts:

$$J(T) = \frac{2}{\pi}\left[0.7854 + \frac{0.8863}{2\sqrt{T}} + 0.2146\, exp\left\{-\frac{0.7823}{2\sqrt{T}}\right\}\right] \qquad \text{[Equation 10]}$$

The concentration profile at a dimensionless distance $\mathrm{P} = \frac{\rho}{r_\mathrm{p}} = \frac{\sqrt{r^2 + (z - z_\mathrm{p})^2}}{r_\mathrm{p}}$ from the centre of a hemisphere on a surface ($Z_\mathrm{p} = z_\mathrm{p}/r_\mathrm{p} = 0$ in the truncated model) from which material releases into solution is given analytically by Crank[13] as

$$C(\mathrm{P},T) = \frac{K_j}{\mathrm{P}(1+K_j)}\Bigg[\mathrm{erfc}\left(\frac{\mathrm{P}-1}{2\sqrt{T}}\right) - \exp\left\{(K_j+1)(\mathrm{P}-1) + (K_j+1)^2 T\right\}\mathrm{erfc}\left(\frac{\mathrm{P}-1}{2\sqrt{T}} + (K_j+1)\sqrt{T}\right)\Bigg] \quad \text{[Equation 11]}$$

where P is the distance from the centre of the sphere, T is the dimensionless time, and K_j is the dimensionless kinetic release rate constant into medium j. The steady-state, diffusion-limited flux from a sphere in point contact with a surface has been derived analytically by Bobbert, Wind and Vlieger[14]:

$$J_{\mathrm{Tot}} = 2\ln 2 \quad \text{[Equation 12]}$$

and the flux-versus-θ profile has been calculated by Streeter[15].

2 Distinctions of disk model from truncated sphere model

The equations used in the truncated sphere model are also applied within the disk model. However, the active surface is changed to a flat disk and the reference length is changed from the particle radius to the disk radius. The values of D and $[\mathrm{A}]_{\mathrm{eq}}$ are also changed; however, because of the conversion to dimensionless coordinates, their dimensionless counterparts are not affected while we simulate the cuticular medium independently from the aqueous. We apply a perfect sink boundary condition at the planar boundary opposite the disk to represent the interface between the 'cuticle proper' and the 'sorption compartment'. The distance between the planes in the z- direction, which represents the thickness of the 'cuticle proper' is given by $Z_{\mathrm{cut}} = z_{\mathrm{cut}}/r_{\mathrm{p}}$ which is equivalent to the Z_{max} of the simulation space.

3 Release from a spherical particle into infinite volume

Simulation results for pesticide release from a spherical particle into an unbounded aqueous medium, for which an analytical equation has been derived by Crank[13] (see Supplementary Information Section 1.7), are presented. The release is presented as a function of K_{aq}, the dimensionless release rate constant into the aqueous medium. The plot in Supplementary Figure 3 shows excellent agreement with analytical theory, demonstrating thermodynamic and kinetic limits.

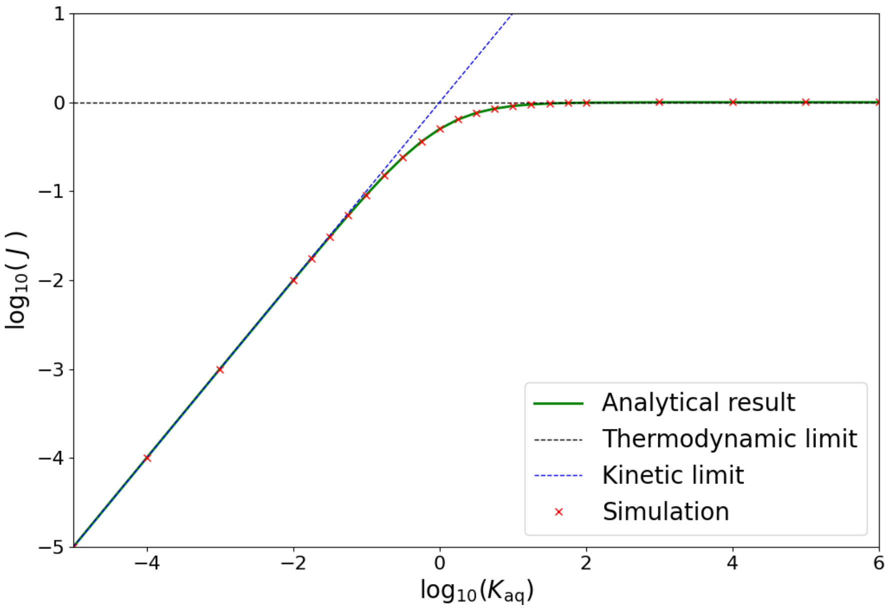

Supplementary Figure 3: The logarithm of the steady-state dimensionless surface flux, J, as a function of the dimensionless release rate constant, K_{aq}, with limiting regimes illustrated, for a sphere in an unbounded medium, with analytical results from Crank's derivation.

These results validate the simulation approach taken and provide insight into the analysis of later simulations with respect to potential limiting cases. Supplementary Figure 4 further illustrates these limiting cases.

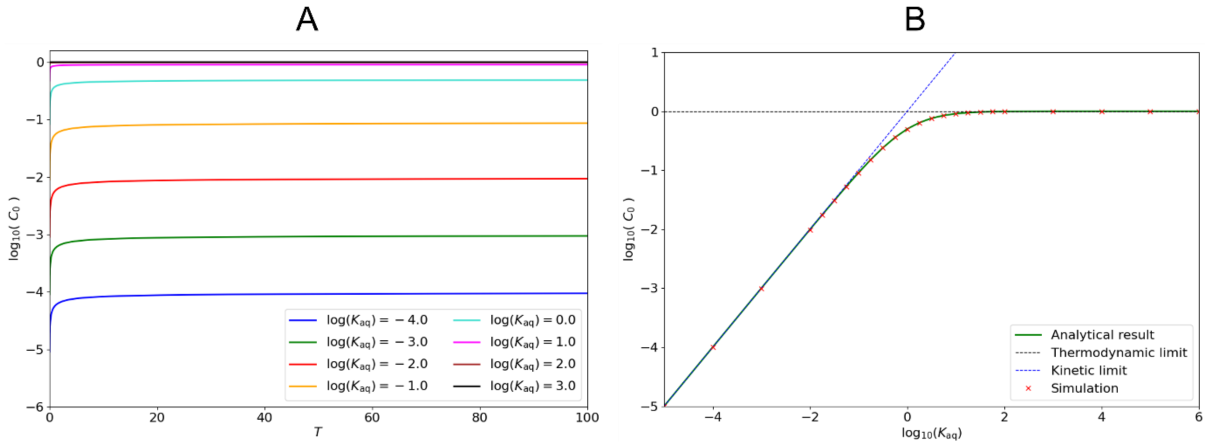

Supplementary Figure 4: Further results for an isolated spherical particle releasing pesticide into an unbounded medium. (A) Dimensionless concentration on the particle surface C_0 as a function of dimensionless time T with varying values of K_{aq} illustrating finite time to attain steady-state result for release under the kinetic limit. (B) Steady-state dimensionless concentration on the particle surface as a function of K_{aq}, illustrating the limits. The analytical result derived by Crank is included as a green solid line. Note that this plot is identical to the plot of steady-state surface flux versus K_{aq}.

4 Flux at the sink boundary for constrained disk

The dependence of the flux at the perfect sink boundary on the release kinetics and relative boundary thickness Z_{cut} is a valid metric for the successful penetration of material through the 'cuticle proper'

barrier. Supplementary Figure 5 illustrates the dependence of the flux on the barrier thickness under thermodynamic (A and C) and kinetic (B and D) release limits.

Plot A illustrates the steady-state flux profile at the perfect sink plane boundary under the thermodynamic release regime at the particle-cuticle interface. One observes that as one enters deeper into the constrained regime, the flux at the sink boundary tends towards the predicted linear flux of $J(R \leq 1) = 1/Z_{\text{cut}}$ and the flux decays increasingly sharply for $R > 1$. Plot C highlights the difference in the sharpness of the decay in flux. As the barrier thickness grows, the rate of diffusion in the z-direction relative to the r-direction is reduced such that the flux profile extends to larger R. Under the steady state, the flux into the system must equal flux out of it, thus the total flux at the plane sink boundary must equal the total flux from the disk. Thus, the principal effect of shortening the barrier thickness is the localization of material within $R \leq 1$.

We perform analogous simulations under the kinetic regime with $K_{\text{cut}} = 10^{-6}$. Plots B and D illustrate a similar trend as was observed for the thermodynamic regime whereby the flux profile tends towards a step for decreasing Z_{cut}. However, the flux produced by linear diffusion is limited by the release kinetics and thus does not scale with the barrier thickness. The total flux remains equal to the total flux at the disk surface: $J_{\text{Tot}}/r_{\text{p}}^2 = \pi K_{\text{cut}}$ for all values of Z_{cut}.

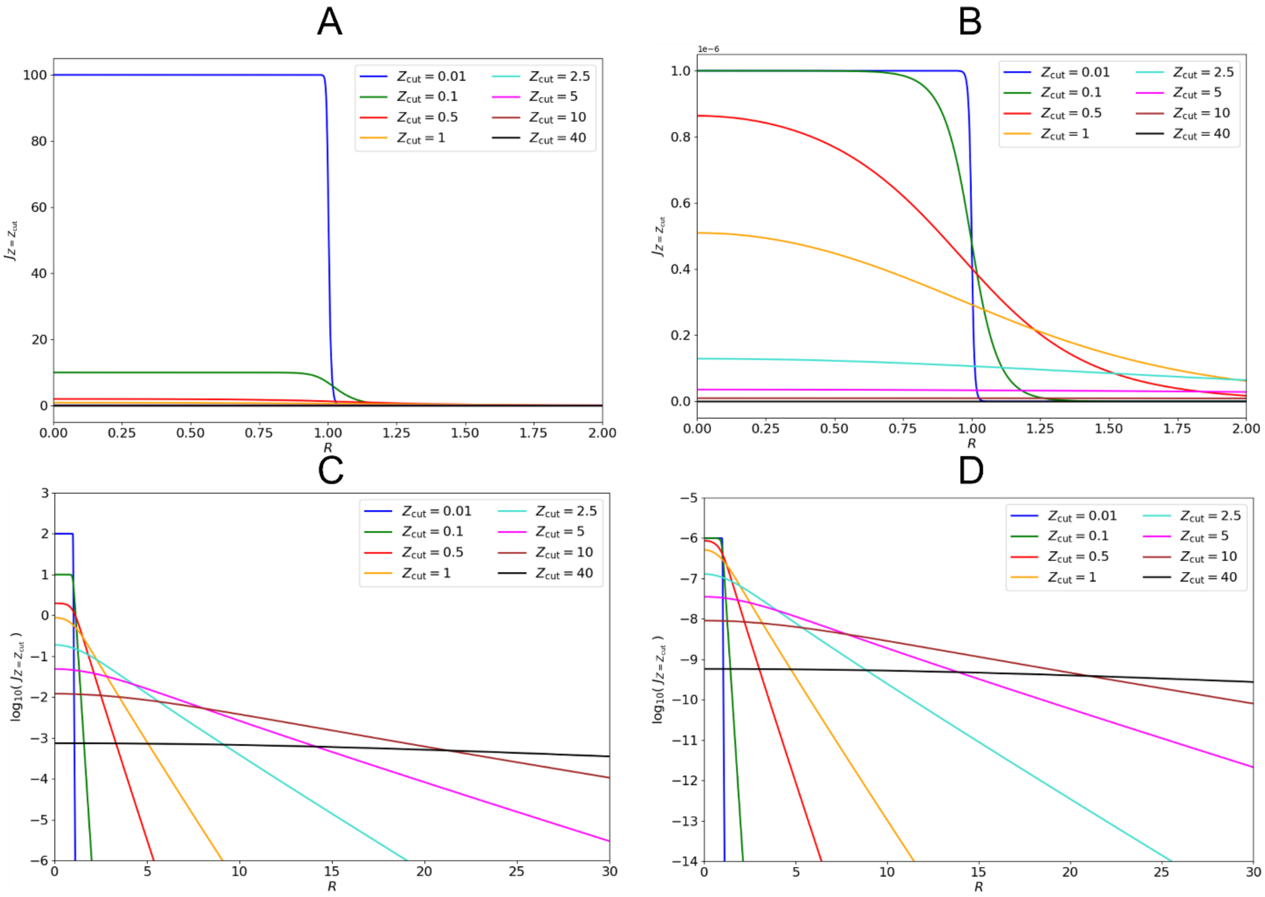

Supplementary Figure 5: Dimensionless steady-state flux profile across the sink plane boundary $J_{Z=Z_{\text{cut}}}$ for varying barrier thicknesses Z_{cut} under (A) thermodynamic release ($K_{\text{cut}} = 10^6$) and (B) kinetic release ($K_{\text{cut}} = 10^{-6}$). Profile of the logarithm of the dimensionless steady-state flux across

the sink plane boundary $\log_{10}(J_{Z=Z_{\mathrm{cut}}})$ for varying barrier thicknesses Z_{cut} under (C) thermodynamic release ($K_{\mathrm{cut}} = 10^{6}$) and (D) kinetic release ($K_{\mathrm{cut}} = 10^{-6}$).

Localization of the flux with respect to the r-dimension may have significant effects on the uptake beyond the 'cuticle proper' by reducing the volume into which material is sequestered and maintaining a larger concentration gradient into the leaf. This requires further study and revision of the perfect sink approximation at the 'cuticle proper'-'sorption compartment' interface. Future study of the flux transient at this far boundary may also be of value for determining time-dependent uptake and lag induced by transport across the membrane barrier.

5 Dependence on time to steady-state disk flux for variable barrier thickness Z_{cut}

Decreasing the value of Z_{cut} also decreases the time taken to achieve steady-state flux. This is illustrated in Supplementary Figure 6. The flux is unchanged by the barrier thickness until the diffusion layer arrives at the sink boundary. Tuning the uptake rate decay towards a constant steady state uptake is significant for the design of formulations for which slow, continuous uptake is preferred from the moment of application. This is achieved sooner for particles with larger contact areas with the cuticle.

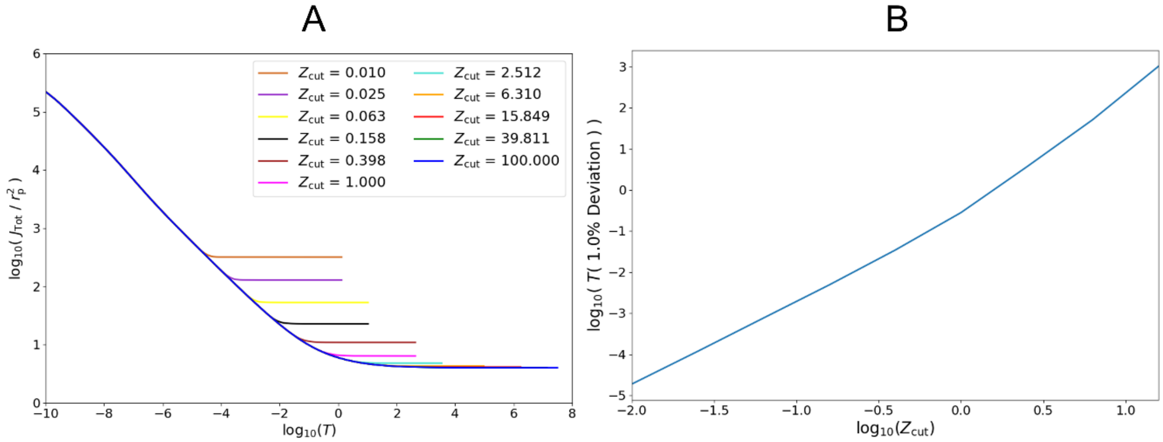

Supplementary Figure 6. (A) Logarithm of the total flux J_{Tot} from a disk into a finite planar barrier under the thermodynamic regime ($K_{\mathrm{cut}} = 10^{6}$) with respect to dimensionless time T for varying relative barrier thicknesses Z_{cut} between constrained and infinite regimes. (B) Logarithm plot of the time taken for the flux transient to deviate from the infinite-volume limit by 1% as a function of Z_{cut}

6 G-parameter: predicting the particle-release regime

We identify the parameter G, where $G = \frac{D_{\mathrm{cut}}[\mathrm{A}]_{\mathrm{eq}}^{\mathrm{cp}}}{2D_{\mathrm{aq}}[\mathrm{A}]_{\mathrm{eq}}^{\mathrm{aq}}Z_{\mathrm{cut}}}$ under the constrained barrier regime or $G = \frac{2D_{\mathrm{cut}}[\mathrm{A}]_{\mathrm{eq}}^{\mathrm{cp}}}{D_{\mathrm{aq}}[\mathrm{A}]_{\mathrm{eq}}^{\mathrm{aq}}}$ if the barrier thickness is within the infinite regime. We can thus conclude that if $K_{\mathrm{aq}} \gg G$, the particle will be fully depleted of pesticide before direct uptake can meaningfully occur. If the aqueous release is slowed, such that direct uptake can act meaningfully before particle depletion, direct and indirect uptake may compete. If $G < 1$, then for direct uptake to occur before the complete depletion of material from the particle, the aqueous release must be slowed to such an extent that only direct uptake can be active. If $G > 1$ then a region in which the two pathways can compete simultaneously is accessible.

$G \ll 1$ throughout the ranges of physicochemical properties considered above for lipophilic pesticides. We predict that foliar pesticide uptake cannot meaningfully occur by both pathways simultaneously and so can be approximated as either direct or indirect uptake acting alone.

7 Supplementary Figures

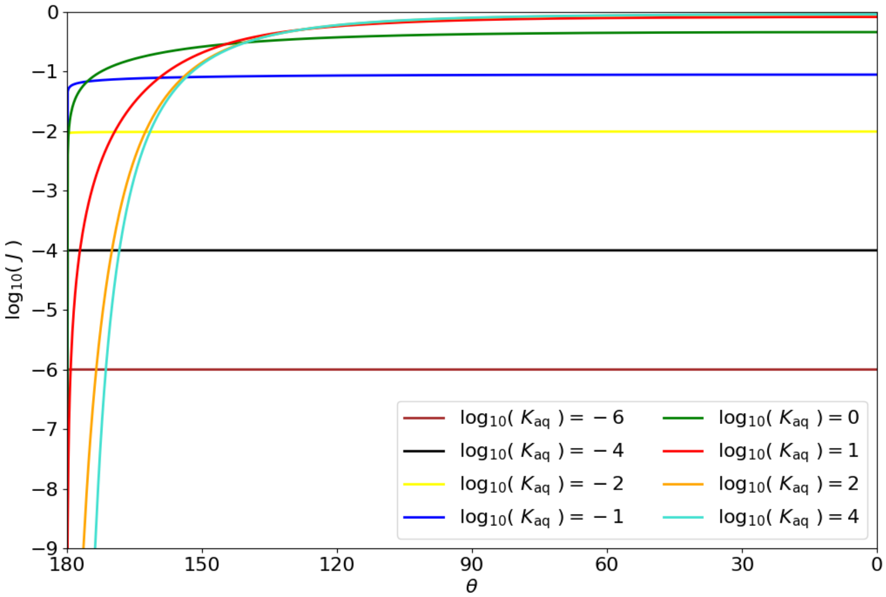

Supplementary Figure 7: Variation of the logarithm of the dimensionless steady-state flux profile J across the surface of the sphere with $Z_{\mathrm{p}} = 1$ for different values of the dimensionless release rate constant K_{aq}, illustrating the thermodynamic and kinetic regimes for a sphere on an inert plane surface.

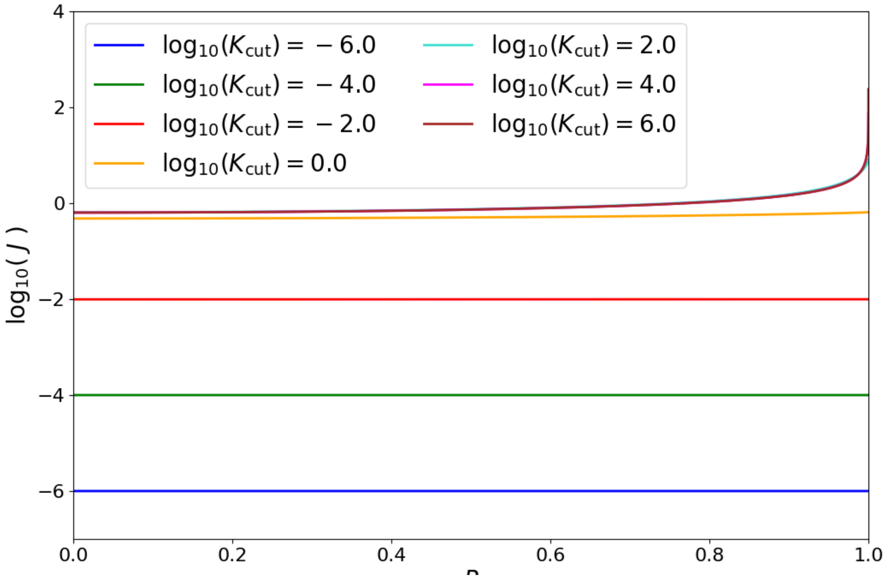

Supplementary Figure 8: Steady-state surface flux profile J across the area of the particle-cuticle contact disk with $Z_{\mathrm{cut}} = 1000$ and varying K_{cut}.

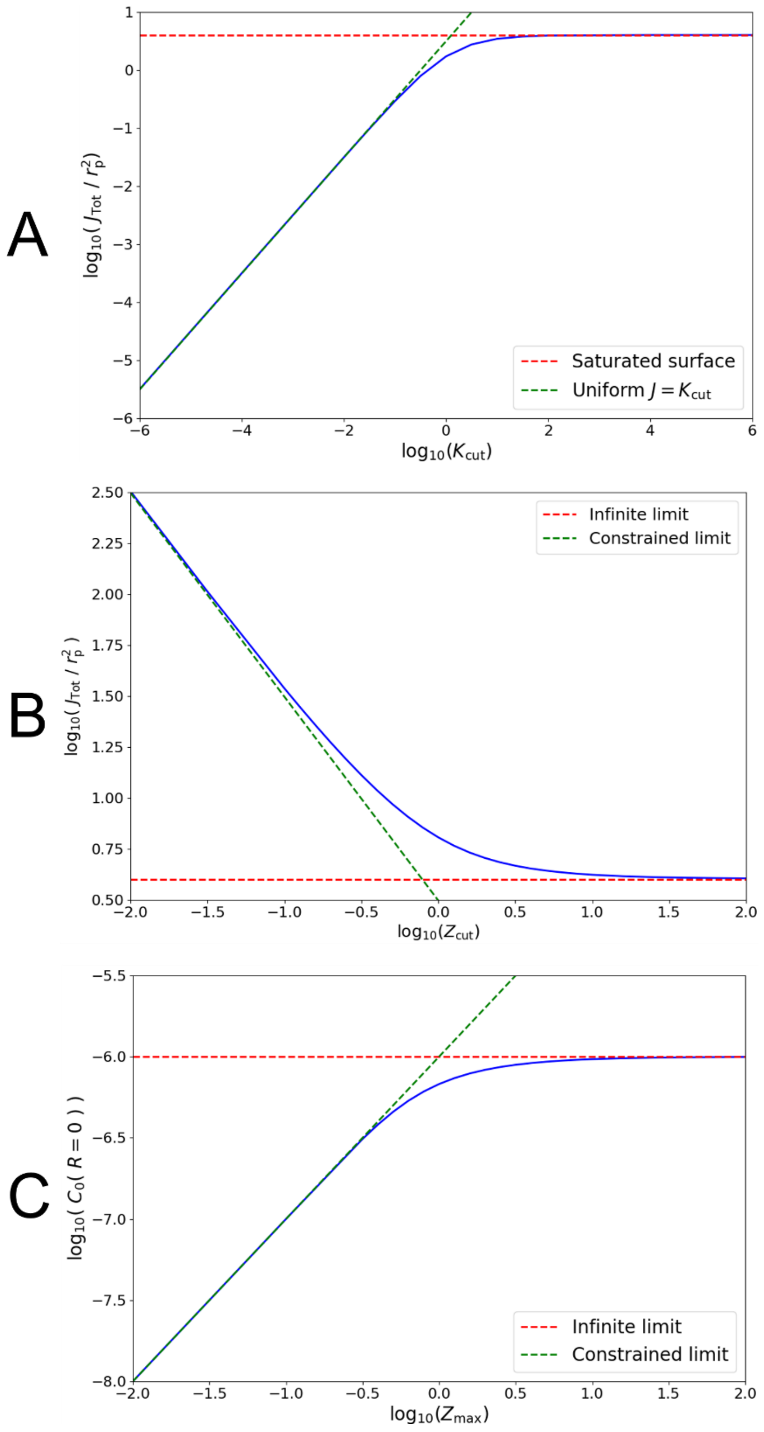

Supplementary Figure 9: Illustration of, for direct uptake from a particle-cuticle disk contact area, (A) the thermodynamic-kinetic regime transition according to the total dimensionless particle-cuticle surface flux J_{Tot} in the infinite barrier limit, (B) the infinite-constrained regime transition according to the total dimensionless particle-cuticle surface flux J_{Tot} in the thermodynamic release limit, and (C) the infinite-constrained regime transition according to the dimensionless surface concentration at the symmetry axis $C_0(R = 0)$ in the kinetic release limit.

8 Supplementary References

1. A. Fick, *The London, Edinburgh, and Dublin Philosophical Magazine and Journal of Science*, 1855, **10**, 30-39.
2. R. G. Compton, E. Kätelhön, E. Laborda and K. R. Ward, *Understanding voltammetry: Simulation of electrode processes*, World Scientific Europe, Second edn., 2020.
3. A. Einstein, *Annalen der Physik*, 1905, **322**, 549-560.
4. M. von Smoluchowski, *Annalen der Physik*, 1906, **326**, 756-780.
5. M. Rudolph, *Journal of Electroanalytical Chemistry*, 2002, **529**, 97-108.
6. D. J. Gavaghan, *Journal of Electroanalytical Chemistry*, 1998, **456**, 1-12.
7. K. R. Ward, N. S. Lawrence, R. S. Hartshorne and R. G. Compton, *Journal of Electroanalytical Chemistry*, 2012, **683**, 37-42.
8. J. A. Alden and R. G. Compton, *The Journal of Physical Chemistry B*, 1997, **101**, 8941-8954.
9. D. J. Gavaghan and J. S. Rollett, *Journal of Electroanalytical Chemistry and Interfacial Electrochemistry*, 1990, **295**, 1-14.
10. D. W. Peaceman and J. Rachford, H. H. , *Journal of the Society for Industrial and Applied Mathematics*, 1955, **3**, 28-41.
11. K. Aoki, *Review of Polarography*, 2017, **63**, 21-28.
12. D. Shoup and A. Szabo, *Journal of Electroanalytical Chemistry and Interfacial Electrochemistry*, 1982, **140**, 237-245.
13. J. Crank, *The Mathematics of Diffusion*, Clarendon Press, 1979.
14. P. A. Bobbert, M. M. Wind and J. Vlieger, *Physica A: Statistical Mechanics and its Applications*, 1987, **141**, 58-72.
15. I. Streeter and R. G. Compton, *The Journal of Physical Chemistry C*, 2007, **111**, 18049-18054.
